# Keratosis Follicularis Spinulosa Decalvans Associated With Woolly Hair: A Case Report

**DOI:** 10.7759/cureus.65668

**Published:** 2024-07-29

**Authors:** Reem Brashi, Raghad Saleh, Ethar A Alsulami, Ahmed Niyazi, Maria AlSulami, Khalid Al Hawsawi

**Affiliations:** 1 College of Medicine, Umm Al-Qura University, Makkah, SAU; 2 Dermatology, King Abdulaziz Hospital, Makkah, SAU

**Keywords:** keratosis pilaris atrophicans, kfsd, scarring alopecia, woolly hair, keratosis follicularis spinulosa decalvans

## Abstract

Keratosis follicularis spinulosa decalvans X-linked (KFSDX) is part of the spectrum of a rare disorder known as keratosis pilaris atrophicans. Here, we report the case of a 14-year-old boy who presented with a history of abnormal hair since birth. He also had a history of skin lesions and hair loss. There was no similar condition in the family, and the parents were not consanguine. Scalp examination revealed woolly hair, a solitary scarring alopecia patch, and follicular papules. There were also patches of scarring alopecia on the lateral portion of the eyebrows and whole eyelashes bilaterally. His nose showed multiple, skin-colored, non-scaly follicular papules. The differential diagnosis included lichen planopilaris, Graham Little-Piccardi-Lassueur syndrome, KFSDX, keratosis follicularis spinulosa decalvans, and structural hair anomalies. Hair examination under light microscopy was normal. Skin biopsy from the follicular papule on the nose revealed follicular plugging with normal epidermis and dermis. Based on the above clinicopathological findings, the patient was diagnosed with KFSDX associated with woolly hair. He was reassured, but he did not show up for further treatment during the follow-up.

## Introduction

Keratosis follicularis spinulosa decalvans X-linked (KFSDX) is part of the spectrum of a rare disorder known as keratosis pilaris atrophicans. It has four variants including KFSDX, keratosis pilaris atrophicans faciei (ulerythema ophryogenes), atrophoderma vermiculata, and keratosis follicularis spinulosa decalvans (KFSD). The latter is an autosomal dominant condition also known as folliculitis spinulosa decalvans (FSD) [1]. These disorders are characterized by inflammatory keratotic follicular papules that later end in atrophy causing cicatricial alopecia [2]. KFSDX is an X-linked inherited disorder that affects the scalp, eyebrows, eyelashes, cheeks, nose, neck, dorsal hands, and fingers, whereas, in ulerythema ophryogenes, the lateral eyebrows, temples, cheeks, and forehead are affected [2]. In atrophoderma vermiculata, the cheeks, neck, and limbs are affected [3]. KFSD or FSD presents with follicular pustules on the scalp along with other features of KFSDX [3]. KFSDX is an X-linked recessive condition caused by a mutation in the membrane-bound transcription factor protease site 2 (*MBTPS2*) gene [4]. The onset of KFSDX starts during childhood with follicular keratotic papules. At puberty, the scarring alopecia develops. Woolly hair has been reported with KFSDX. The patient described in this case showed this very rare association of woolly hair with KFSDX.

## Case presentation

**Figure 1:**
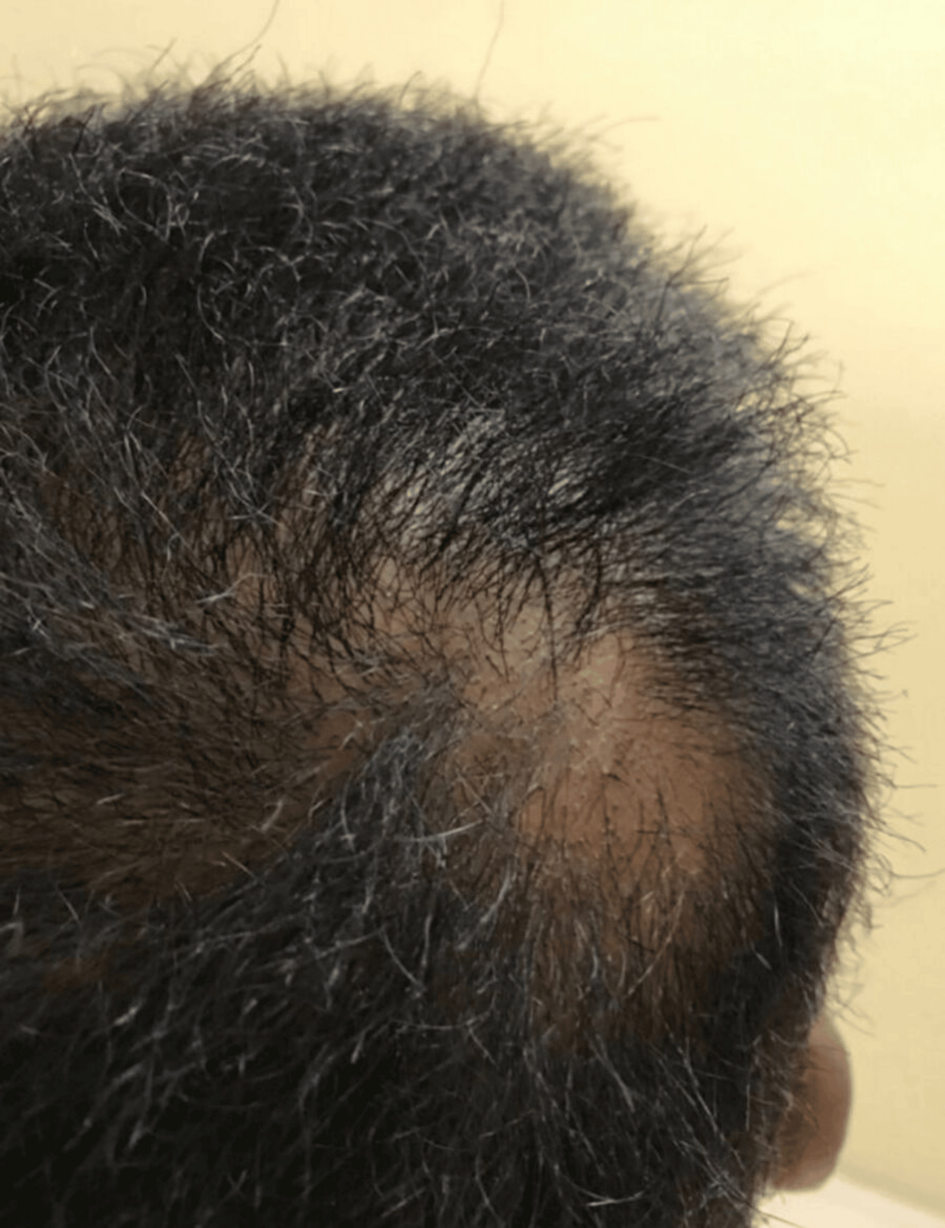
Scarring alopecia and follicular papules.

**Figure 2:**
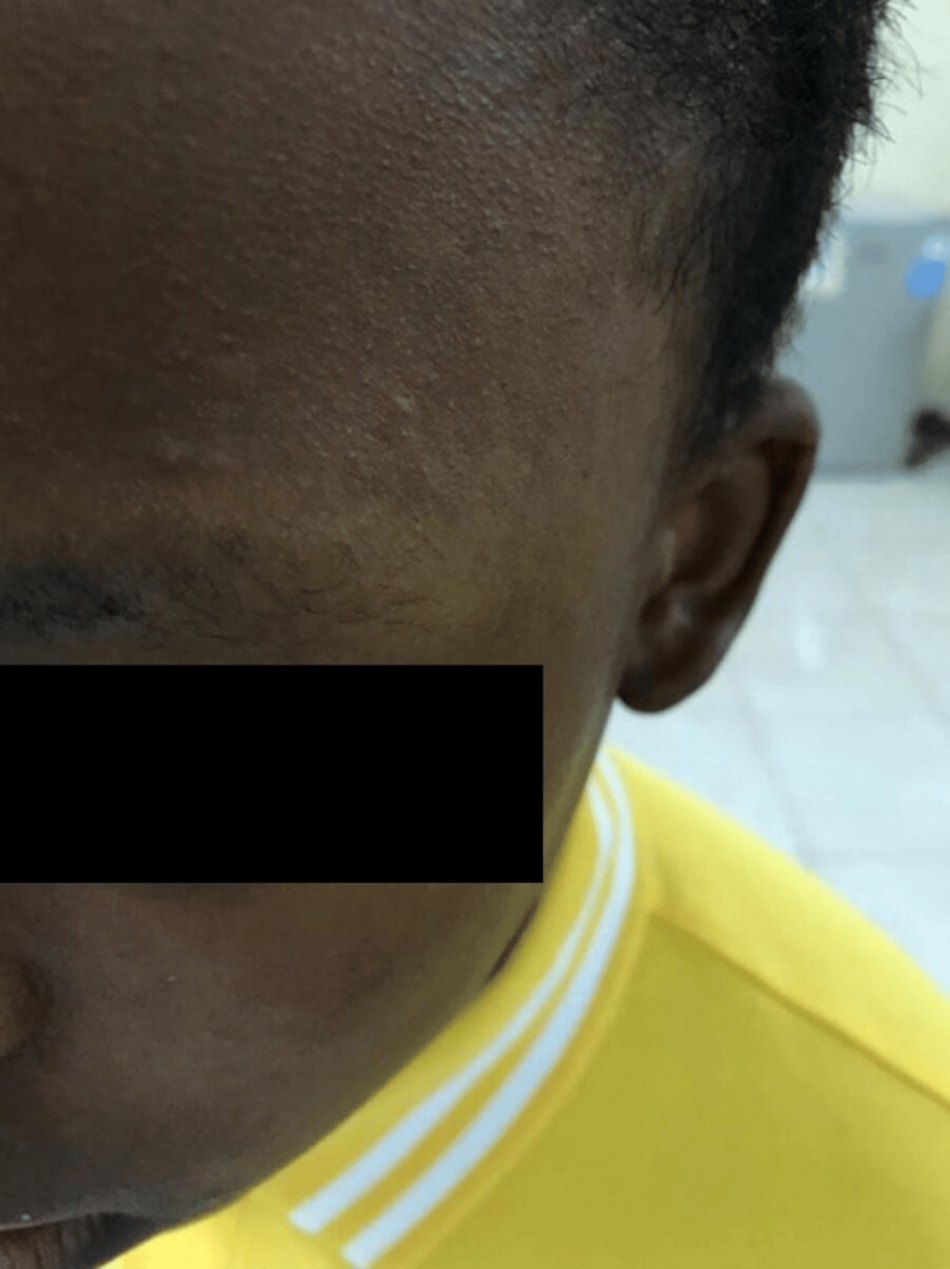
Scarring alopecia over the eyebrow and eyelashes.

A 14-year-old boy presented with a history of abnormal hair since birth. He also had a history of skin lesions for six years, along with a history of hair loss for two years. The review of systems and past medical history was unremarkable. There was no similar condition in the family, and the parents were not consanguineous. Skin examination of the scalp revealed woolly hair as well as the presence of a solitary patch of scarring alopecia with follicular papules (Figure 1). There were also patches of scarring alopecia on the lateral portion of the eyebrows and entire bilateral eyelashes (Figure 2).

**Figure 3:**
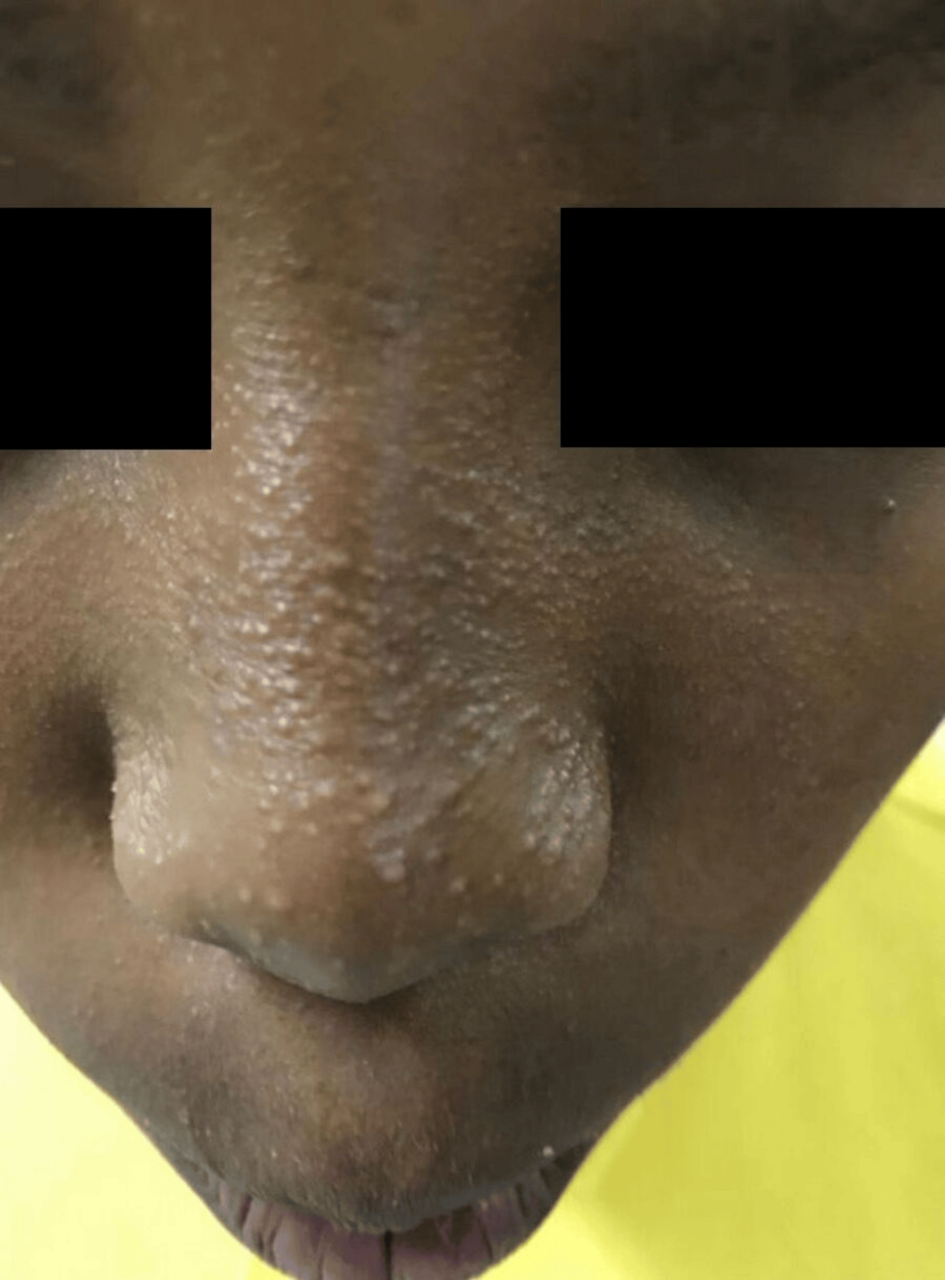
Follicular papules over the nose.

**Figure 4:**
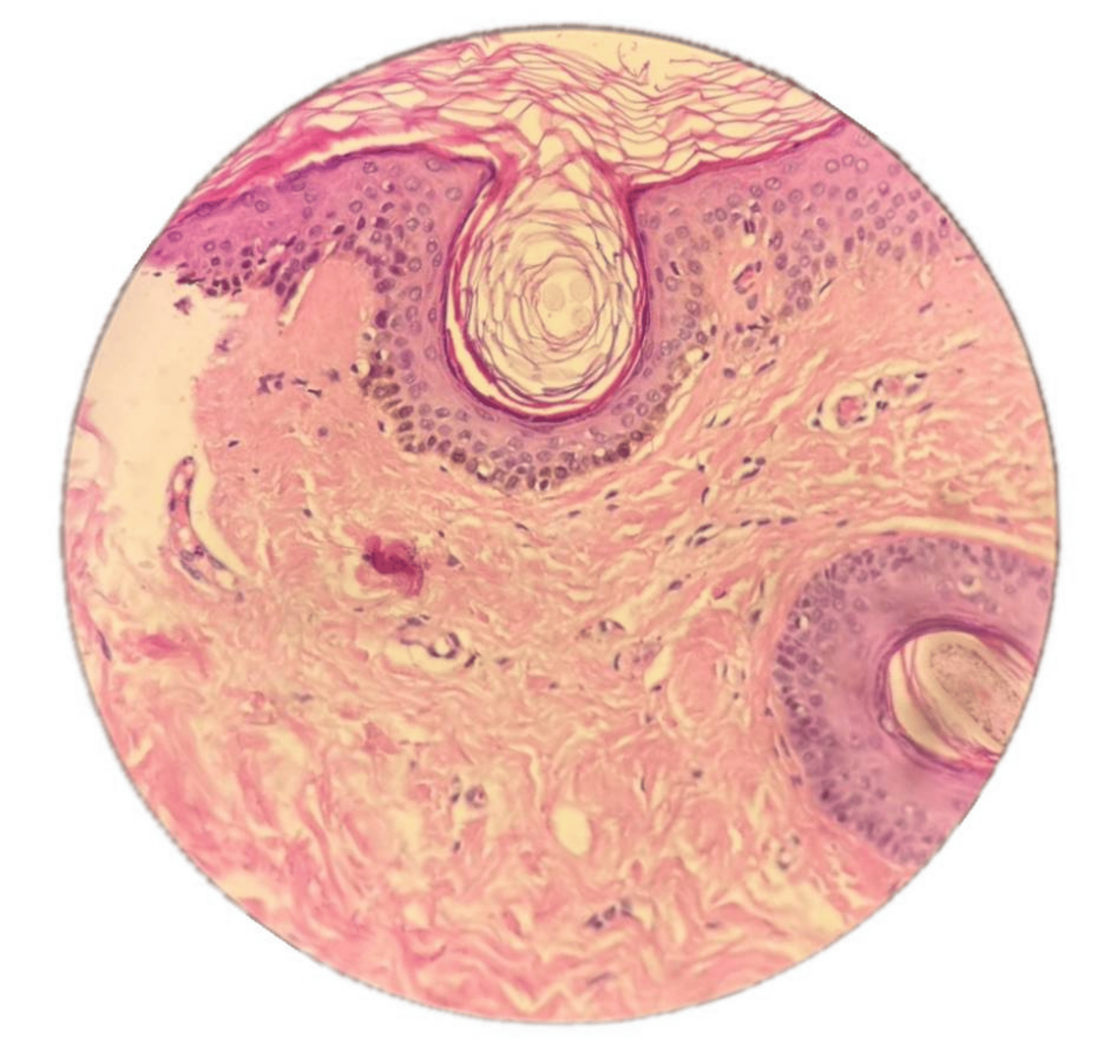
Punch skin biopsy from the follicular papules on the nose showing follicular blugging, but otherwise normal skin.

Additionally, there were multiple skin-colored, non-scaly follicular papules over his nose (Figure 3). The parents were examined, and there were no similar findings. The differential diagnosis included lichen planopilaris (LPP), Graham Little-Piccardi-Lassueur syndrome (GLPLS), KFSDX, KFSD, and structural hair anomalies. Examination of the mucous membranes, palms, and soles was normal. Hair examination under light microscopy was normal. A 3-mm punch skin biopsy was taken from the follicular papule on the nose. It revealed follicular plugging in the normal epidermis and dermis (Figure 4). Based on the above clinicopathological findings, the patient was diagnosed with KFSDX associated with woolly hair. He was reassured. However, he did not show up for further treatment during the follow-up.

## Discussion

KFSDX is a rare X-linked recessive condition that was initially reported by Siemens in 1926. It has been mapped to chromosome Xp22.13-p22.2 with a mutation in the *MBTPS2* gene. Males are primarily affected by this condition [1]. Female carriers can have severe symptoms, but often do not exhibit any disease or have a mild variant [2].

Characteristic features of KFSDX include keratotic follicular papules on the scalp, brows, and eyelashes, which lead to scarring alopecia. It can also manifest as extensive keratosis pilaris on the face, extremities, and trunk, starting in early childhood. Cicatricial alopecia of the scalp develops around puberty and progressively deteriorates. Compared to KFSDX, KFSD has more severe scalp inflammation with pustules. KFSD is similar to KFSDX. However, our patient had no pustules [5].

Additional features associated with KFSDX include acne keloidalis nuchae and tufted hair folliculitis, corneal dystrophy, blepharitis, conjunctivitis, keratitis, photophobia, congenital glaucoma, cataracts, atopy, facial erythema, hyperkeratosis of the palms and soles, cuticular hypertrophy, mental retardation, and developmental delay [3,4]. KFSDX and ichthyosis follicularis with atrichia and photophobia (IFAP) syndrome are allelic and result from mutations in a zinc metalloprotease (*MBTPS2*) that is important for cholesterol homeostasis. While IFAP usually presents with features similar to KFSDX, the alopecia in IFAP is non-scarring. Some studies have considered these two conditions to be a spectrum of one condition [6,7].

The main differential diagnoses for our patient included LPP, GLPLS, and structural hair anomalies. LPP is rare in children. GLPLS primarily affects middle-aged women and is characterized by the non-cicatricial loss of pubic and axillary hair, which was not observed in our patient. The histopathological findings in our patient were consistent with KFSD and not LPP or GLPLS. The limitation in our case was the unavailability of a genetic study. However, although KFSDX has a known gene mutation, KFSD does not have an identified gene mutation yet.

Woolly hair is a genetic disorder characterized by thick, wool-like hair that is often highly pigmented. It is inherited as an autosomal dominant or recessive feature [8,9]. Woolly hair has been reported with ulerythema ophryogenes, KFSD, Naxos disease, Carvajal syndrome, and skin fragility/woolly hair syndrome [5].

Furthermore, KFSDX has been linked to various disorders, including cutis laxa, big pinnae, clinodactyly, arachnodactyly, Noonan’s syndrome, deafness, aminoaciduria, mental retardation, Down’s syndrome, congenital glaucoma, lenticular cataract, hepatomegaly, and bilateral inguinal hernia [2,3,7]. However, none of these additional conditions were found in our patient.

There is no known effective treatment for KFSDX [10]. However, certain interventions such as keratolytics, topical or intralesional corticosteroids, topical calcineurin inhibitors, topical or systemic retinoids, phototherapy, and dapsone may help reduce the keratotic and inflammatory components [5,10].

Some cases in the early stages with significant inflammation have shown quite an improvement with the treatment, although overall the results are often unsatisfactory [4]. In our case, the patient was reassured, but he did not show up during follow-up for further treatment.

## Conclusions

We presented the case of a 14-year-old boy with scarring alopecia on the scalp, eyebrows, and eyelashes. He also had skin-colored follicular papules on his nose. All of these features can be seen in both LPP and KFSDX. The crucial feature that distinguishes LPP from KFSDX is the histopathological features. LPP has a distinguished histopathology which was not seen in our patient.
